# Association between systemic inflammation biomarkers and outcomes in patients with aneurysmal subarachnoid hemorrhage

**DOI:** 10.3389/fneur.2026.1749871

**Published:** 2026-03-17

**Authors:** Maria Della Giovampaola, Andrea Vieno, Adeline Higuet, Irene Cavalli, Claudia Stella, Giacomo Coppalini, Alberto Diosdado, Daniel Damasceno, Lucas Freitas, Fernando Oliveira Gomes, Ana Carolina Damasceno Lacerda Fernandes, Marcos Vinicius Tavares De Magalhães, Vinicius William Costa Dumont, Fabio Silvio Taccone, Elisa Gouvêa Bogossian

**Affiliations:** 1 Department of Intensive Care, Hôpital Universitaire de Bruxelles (HUB), Université Libre de Bruxelles (ULB), Brussels, Belgium; 2 Department of Medical and Surgical Sciences, Alma Mater Studiorum, University of Bologna, Bologna, Italy; 3 Anaesthesia and Intensive Care Unit, University Hospital Integrated Trust of Verona, Verona, Italy; 4 Department of Emergency Medicine, Hôpital Universitaire de Bruxelles (HUB), Université Libre de Bruxelles (ULB), Brussels, Belgium

**Keywords:** biomarkers, inflammation, ischemia, medical complications, outcome, stroke

## Abstract

Aneurysmal subarachnoid hemorrhage (aSAH) is a devastating condition that is associated with cerebral and systemic inflammation. C-reactive protein (CRP) and neutrophil-to-lymphocyte ratio (NLr) are easily available biomarkers of systemic inflammation. Therefore, we aimed to assess the impact of elevated CRP and NLr on aSAH outcomes. This retrospective, single-center study included adult patients admitted with aSAH to the intensive care unit (ICU) from January 2007 to December 2023. We recorded serum CRP and NLr levels during the first 7 days of ICU stay. An unfavorable neurological outcome at 3 months was defined as a Glasgow Outcome Scale (GOS) score of 1–3. A total of 547 patients were included in the study; 250 (45.7%) experienced unfavorable outcomes (UOs), and 140 (25.6%) developed delayed cerebral ischemia (DCI). Patients with unfavorable outcomes had higher levels of CRP from days 2 to 7 after SAH (*p* = 0.001) and higher NLr (*p* = 0.06) than those with favorable outcomes (FOs). In a multivariate logistic regression model, the highest CRP value (OR: 1.003, 95% CI: 1.001–1.005) and the highest NLr value (OR: 1.025; 95% CI: 1.001–1.050) in the first 7 days after SAH were independently associated with the occurrence of unfavorable outcomes. The highest NLr value was also associated with the development of DCI (sHR: 1.02, 95% CI: 1.01–1.03). In conclusion, high CRP and NLr values have a significant prognostic role in aSAH patients, reinforcing the importance of inflammation as a potential mechanism of secondary brain injury.

## 1 Introduction

Spontaneous subarachnoid hemorrhage (SAH) is a devastating condition that accounts for 5% of all strokes and affects 7.9 per 100,000 person-years (1, 2). Immediately after blood is released into the subarachnoid space, ventricles, and parenchyma, there is a significant rise in intracranial pressure (ICP), acute vasoconstriction, and microthrombosis, leading to a reduction in cerebral blood flow (CBF) and promoting cerebral edema (3). Ischemia combined with reactive oxygen species and toxic hemoglobin breakdown products activates the inflammatory cascade, causing neuroinflammation and blood–brain barrier dysfunction, which further aggravates cerebral edema (4, 5).

Neuroinflammatory cascades are primarily mediated by resident microglial cells and infiltrating leukocytes (6). Leukocytic infiltration from the peripheral blood is one of the first inflammatory events, as these cells stimulate the microglia and activate innate responses by secreting pro-inflammatory cytokines (7). Central cytokine expression by glial cells further promotes the recruitment of neutrophils, monocytes, macrophages, and other immune cells (8). Moreover, degraded blood products stimulate toll-like receptors (TLR-4) (9), enhancing inflammatory cytokine production and perpetuating intrinsic inflammatory pathways (7).

Interestingly, SAH is also associated with intense systemic inflammation, involving complex interactions between the coagulation cascade (10), sympathetic activation (11), and endothelial dysfunction (12). Patients often exhibit clinical signs of systemic inflammatory response syndrome (SIRS), such as fever, tachypnea, tachycardia, and leukocytosis (13), as well as organ dysfunction (14). The presence of SIRS in SAH patients has been associated with secondary brain injury, delayed cerebral ischemia (DCI), and worse outcomes (15, 16). Additionally, SAH patients also experience a decrease in lymphocytes (17), particularly T-cell regulators, contributing to a state of induced immunodepression (18).

In this context, serum biomarkers of inflammation may be useful for assessing the presence of a systemic inflammatory response, potentially identifying patients at high risk of DCI and unfavorable neurological outcomes after SAH. The neutrophil-to-lymphocyte ratio (NLr) is a readily available biomarker that reflects inflammatory disturbances by demonstrating a combination of significant neutrophilia with relative lymphopenia. Indeed, an elevated NLr appears to be associated with DCI and poor outcomes after SAH (19). Of note, the majority of studies have focused on one-time measurement of the NLr, usually on admission (20, 21). C-reactive protein (CRP) is a well-known acute-phase inflammatory protein involved in the classical complement pathway of innate immunity (22). In SAH, some studies have reported an association between elevated CRP and unfavorable neurological outcomes (23–30), whereas others have not (31–33). Conflicting results have also been reported regarding the association between elevated CRP and the development of DCI (31–35).

Therefore, we aimed to assess the association of CRP and NLr during the first 7 days after SAH with the occurrence of unfavorable neurological outcomes and the development of DCI.

## 2 Methods

### 2.1 Study design and population

This is a retrospective, single-center cohort study of consecutive aneurysmal SAH (aSAH) patients admitted to the Intensive Care Unit (ICU) of the “Hôpital Universitaire de Bruxelles” (HUB) in Brussels, Belgium, between January 2007 and August 2023. We included all consecutive adult (>18 years old) patients with a diagnosis of ruptured aneurysm as the primary cause of SAH, confirmed by neuroimaging [CT angiography (CTA) and/or digital subtraction angiography (DSA)]. We excluded patients who stayed less than 24 h in the ICU, patients without admission values for CRP and blood cell counts, and patients with pre-existing immunosuppression (e.g., human immunodeficiency virus (HIV), chronic steroid use, or chronic immunosuppressant use). This study was approved on 5 July 2024 by the Ethics Committee of Erasme Hospital (P2024/289), which waived the need for informed consent. All methods were carried out in accordance with the guidelines and regulations of the Declaration of Helsinki. This study was performed according to the STrengthening the Reporting of OBservational Studies in Epidemiology (STROBE) guidelines (36).

### 2.2 Data collection

Demographic and clinical data, including age, sex, and comorbidities, were recorded. Neurological status on admission was assessed using the World Federation of Neurological Surgeons score (WFNS) (37) and Glasgow Coma Scale (GCS) (38). The severity of bleeding was assessed using the modified Fisher scale (mFisher) (39). The Acute Physiology and Chronic Health Evaluation II (APACHE II) (40) score and the Sequential Organ Failure Assessment (SOFA) (41) score were calculated on admission. Aneurysm location and treatment (e.g., coiling and/or clipping) were also collected. We recorded neurological complications, such as rebleeding, intracranial hypertension, angiographic vasospasm, DCI, hydrocephalus, and seizures, as well as their management (i.e., osmotic therapy, decompressive craniectomy, and intra-arterial vasodilators), as previously reported (42). Vasospasm was defined by a neuroradiologist as moderate-to-severe arterial narrowing (>50%) on specific imaging (DSA or CTA) not attributable to atherosclerosis, catheter-induced spasm, or vessel hypoplasia (43). Cerebral vasospasm could also be defined using transcranial Doppler assessment as mean flow velocity in any vessel >200 cm/s or >120 cm/s with a Lindegaard ratio above 3 (44). DCI (45) was defined as the occurrence of focal neurological impairment or a decrease of at least 2 points on the GCS that lasted for at least 1 h, was not apparent immediately after aneurysm occlusion, and could not be attributed to other causes. Additionally, the presence of a new cerebral infarction on a CT or magnetic resonance (MR) scan of the brain within 6 weeks after SAH or proven at autopsy, not attributable to other causes, and not present on the admission CT scan or immediately after aneurysm occlusion was also considered DCI (45). Rebleeding was defined as sudden clinical deterioration with signs of a new or increased hemorrhage on a CT scan compared with prior CT imaging (46). Hydrocephalus was defined as a bicaudate index above the 95th percentile for age, assessed by an experienced neuroradiologist (47).

The use of oral nimodipine, sedation, vasopressors, inotropic agents, and continuous renal replacement therapy (CRRT) was also recorded. We also documented the development of infection within the first 7 days after admission, using definitions from the Centers for Disease Control and Prevention/National Healthcare Safety Network (CDC/NHSN) (48).

Serum CRP values and neutrophil and lymphocyte counts were collected at admission and then daily for the following 7 days. A CRP level of >10 mg/L was considered “elevated,” according to local laboratory thresholds. No specific cutoff has been established for NLr.

Neurological status was assessed using the Glasgow Outcome Scale (GOS) (49) at 3 months. In our institution, GOS is routinely recorded in the medical chart at 3 months by neurologists or neurosurgeons during the follow-up clinic. A GOS score of 1–3 (death, persistent vegetative state, and severe disability) was considered an unfavorable outcome (UO), while a GOS score of 4 (moderate disability) or 5 (good recovery) was considered a favorable outcome (FO).

### 2.3 Endpoints

The primary endpoint of the study was the association of CRP and NLr with the occurrence of UOs. The secondary endpoints included: (a) the association of CRP and NLr with infections occurring within the first 7 days post-ictus and (b) the association of CRP and NLr with the occurrence of DCI.

### 2.4 Statistical analysis

Continuous data were expressed as mean [standard deviation (SD)] or median [interquartile range (IQR)] according to data distribution. Differences between groups were assessed using Student’s *t*-test or the Mann–Whitney *U*-test for normally or non-normally distributed data for independent samples. For related samples, we used the Friedman test to compare continuous variables, including CRP and NLr. Categorical data were presented as numbers [percentage, (%)], and comparisons between groups were performed using the chi-squared test. We performed univariate and multivariable logistic regression analyses to assess the association between the highest NLr/CRP and UOs, adjusting for pre-specified covariates identified using the “historical method” (50), such as commonly described variables associated with UOs in the literature (e.g., age, DCI, a WFNS score of 4–5, intracranial hypertension, rebleeding, and nimodipine prophylaxis) (1). The independence of errors, the presence of multicollinearity, and the presence of influential outliers were assessed, and none were violated. The results of the multivariate logistic regression models were expressed as odds ratios (ORs) with 95% confidence intervals (CIs). We also performed a competing risk analysis to assess the association between the highest NLr, the highest CRP, and DCI, adjusted for mFisher, the presence of angiographic cerebral vasospasm, a WFNS score of 4–5 (51), age, sex, and history of systemic hypertension. Death was considered a competing factor. The results of the competing risk analysis were presented as subhazard ratios (sHRs) with 95% CIs. We performed a multilevel mixed model with random intercepts at the patient and time level (measured in days) to evaluate the association between repeated measures of NLr/CRP and UOs, as well as the occurrence of any infection within the first 7 days post-bleeding. Only CRP/NLr values that preceded the onset of infection were included in their respective analyses. We performed a sensitivity analysis to account for the interaction between NLr/CRP and infection. A logistic regression analysis was used to assess the impact of CRP and NLr on UOs, adjusting for age, DCI, a WFNS score of 4–5, intracranial hypertension, rebleeding, and nimodipine prophylaxis in patients with and without infection. The independence of errors, the presence of multicollinearity, and the presence of influential outliers were checked, and none were violated. A multilevel mixed model with random intercepts at the patient and time levels (in days) was performed to assess the association of repeated measures of CRP and NLr over time and UOs in patients with and without infection. Statistical significance was considered at a *p*-value of <0.05. No imputation for missing values was performed, as the number of missing NLr and CRP values was less than 10%, and all patients had at least 3 values of NLr and CRP. All analyses were performed using IBM SPSS Statistics version 29 and GraphPad Prism 10.

## 3 Results

### 3.1 Study population

A total of 567 consecutive aSAH patients were admitted during the study period; 20 (3.5%) patients were excluded (*n* = 5, ICU stay less than 24 h; *n* = 15, lack of blood count and CRP levels on admission), resulting in 547 (96.5%) patients included in the final analysis. The characteristics of the study population are shown in Table 1. Patients were predominantly female (61.8%) with a median age of 54 years (IQR: 45–63). The median GCS score on admission was 13 (4–15); 249 (45.5%) patients presented with a WFNS grade of 4–5 (poor grade), and 90.3% of patients had an mFisher score of 3–4. The majority of patients underwent aneurysm endovascular coiling (*n* = 480, 87.8%). In the first 7 days following SAH, 149 (27.2%) patients experienced an infection (mostly pneumonia in 80/149, 53.7%). DCI occurred in 140 (25.6%) patients, and UOs were observed in 250 patients (54.3%).

**Table 1:** Characteristics of the studied population at admission.

Variables	All patients (*n* = 547)	Favorable outcome (*n* = 297)	Unfavorable outcome (*n* = 250)	*p*-value
Age (years), median (IQR: 25–75%)	54 (45; 62.8)	52 (43; 59)	57 (49; 67)	0.001
Female, *n* (%)	338 (61.8)	120 (40.4)	161 (64.4)	0.253
APACHE II, median (IQR: 25–75%)	12 (7; 18.5)	8 (5; 12)	18 (13; 21)	0.001
SOFA score, median (IQR: 25–75%)	4 (1; 8)	2 (1; 4)	8 (5; 9.8)	0.001
GCS, median (IQR)	13 (4; 15)	15 (13; 15)	5 (3; 13)	0.001
mFisher score of 3–4, *n* (%)	494 (90.3%)	253 (85.2%)	241 (96.4)	0.001
WFNS score of 4–5, *n* (%)	249 (45.5%)	65 (21.9%)	184 (73.6)	0.001
Nimodipine	475 (86.8)	284 (95.6)	191 (76.4)	0.001
Aneurysm treatment, *n* (%)				0.009
Endovascular treatment of aneurysm	480 (87.8)	271 (91.2)	209 (83.6)	
Surgical treatment of aneurysm	67 (12.2)	26 (8.8)	41 (16.4)	
Comorbidities, *n* (%)				
Arterial hypertension	231 (42.2)	130 (43.8)	101 (40.4)	0.434
Diabetes	45 (8.2)	14 (4.7)	31 (12.4)	0.002
Heart disease	63 (11.5)	27 (9.1)	36 (14.4)	0.060
Previous ND	38 (7)	18 (6.1)	20 (8)	0.403
Chronic renal failure	9 (1.7)	5 (1.7)	4 (1.6)	0.990
COPD	47 (8.6)	24 (8.1)	23 (9.2)	0.650
Cancer	26 (4.8)	13 (4.4)	13 (5.2)	0.691
Liver cirrhosis	7 (1.3)	2 (0.7)	5 (2)	0.256
ICU management, *n* (%)				
Vasopressors	305 (55.8)	91 (30.6)	214 (85.6)	0.001
Inotropic agents	89 (16.3)	19 (6.4)	70 (28)	0.001
Mechanical ventilation	316 (57.8)	87 (29.3)	229 (91.6)	0.001
Renal replacement therapy	2 (0.4)	0 (0)	2 (0.8)	0.208
Complications, *n* (%)				
Seizures	128 (23.4)	53 (17.9)	75 (30)	0.001
Rebleeding	37 (6.8)	6 (2)	31 (12.4)	0.001
Hydrocephalus	185 (33.8)	71 (23.9)	114 (45.6)	0.001
Cerebral vasospasm	218 (39.9)	115 (38.7)	103 (41.2)	0.540
Delayed cerebral ischemia	140 (25.6)	39 (13.1)	101 (40.4)	0.001
Intracranial hypertension	216 (39.5)	45 (15.2)	171 (68.4)	0.001
Anti-seizure prophylaxis	377 (68.9)	191 (64.3)	186 (74.4)	0.009
Treatment for complications, *n* (%)				
Hypothermia	50 (9.1)	3 (1)	47 (18.8)	0.001
Decompressive craniectomy	29 (5.3)	5 (1.7)	24 (9.6)	0.001
Barbituric coma	73 (13.4)	3 (1)	70 (28)	0.001
Hyperventilation	166 (30.4)	21 (7.1)	145 (58)	0.001
Osmotic therapy	157 (28.7)	21 (7.1)	136 (54.4)	0.001
IA spasmolytic for vasospasm	96 (17.6)	32 (10.8)	64 (25.6)	0.001
Angioplasty for vasospasm	47 (8.6)	22 (7.4)	25 (10)	0.289
Induced hypertension for DCI	155 (28.3)	50 (16.8)	105 (42)	0.001
Infections, *n* (%)				
All	149 (27.2)	55 (18.5)	94 (37.8)	0.001
Pneumonia	80/149 (53.7)	21/55 (38.2)	59/94 (62.8)	0.004
Urinary tract infection	42/149 (28.2)	19/55 (34.5)	23/94 (24.5)	0.164
BSI	20/149 (13.4)	14/55 (25.5)	6/94 (6.4)	0.01
CNS infection	5/149 (3.4)	1 (1.8)	4/94 (4.3)	0.43
Others	2/149 (1.3)	0 (0)	2/94 (2.1)	0.90
Length of stay—days, median (IQR: 25–75%)				
ICU	7 (2; 16)	6 (3; 14)	9 (2; 18)	0.149
Hospital	18 (9; 28)	19 (14; 27)	11 (2; 35)	0.001

Data are presented as counts (%), mean (±SD), or median (IQRs). An unfavorable outcome was defined as a Glasgow Outcome Scale score of 1–3. APACHE, Acute Physiology and Chronic Health Evaluation; SOFA, Sequential Organ Failure Assessment; GCS, Glasgow Coma Scale; ICU, intensive care unit; MV, mechanical ventilation; WFNS, World Federation of Neurosurgical Societies; DM, diabetes mellitus; ND, neurological disease; CRF, chronic renal failure; COPD, chronic obstructive pulmonary disease; RRT, renal replacement therapy; ECMO, extracorporeal membrane oxygenation; EVD, external ventricular drain; ICP, intracranial pressure; cEEG, continuous electroencephalogram; DCI, delayed cerebral ischemia; ICHT, intracranial hypertension; IA, intra-arterial; BSI, blood stream infection; CNS, central nervous system.

### 3.2 C-reactive protein and neutrophil-to-lymphocyte ratio values

**Figure 1:**
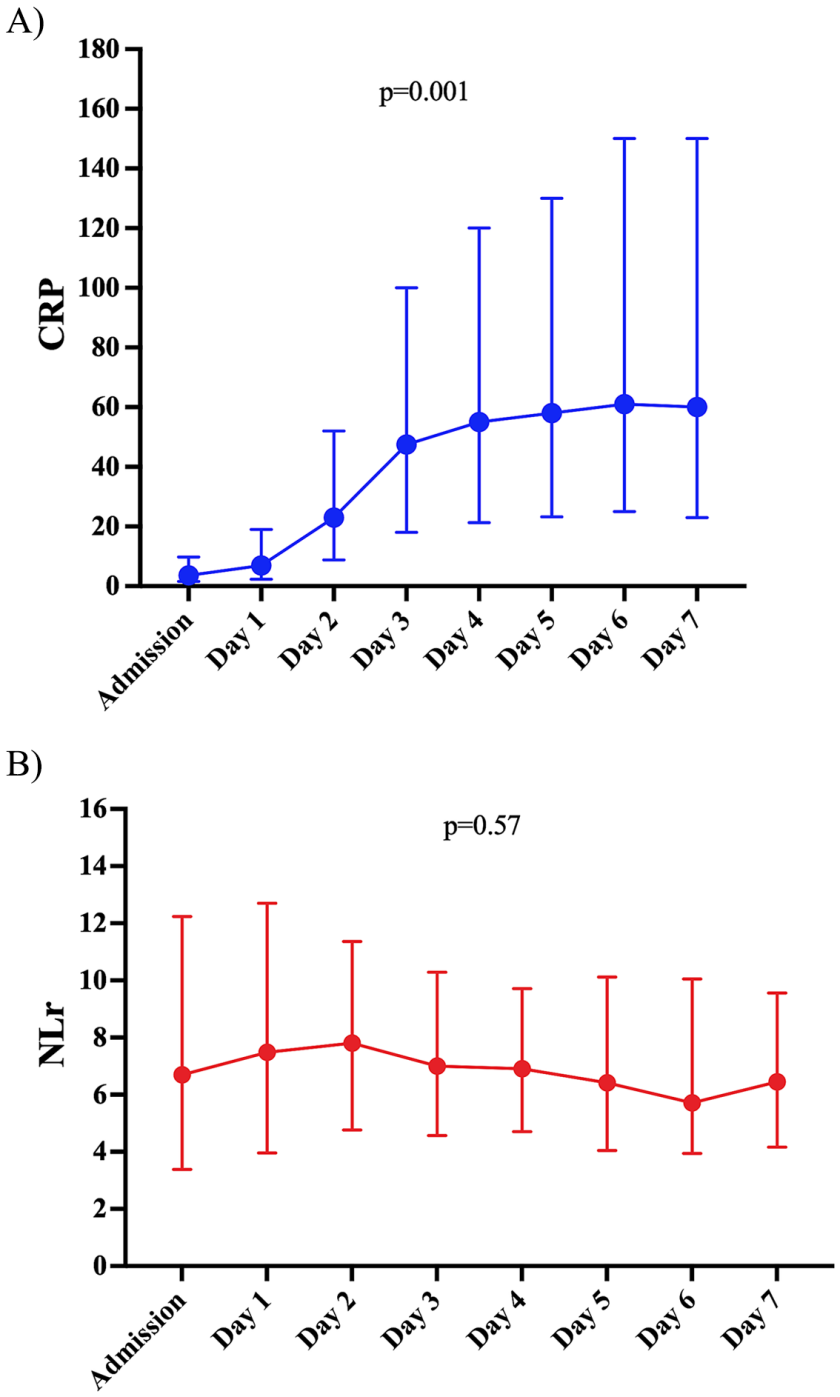
**(A)** C-reactive protein (CRP) levels from admission to the seventh day post-ictus in the study population. Data are presented as a median and interquartile range of 25–75%. The *p*-value was calculated using the Friedman test. **(B)** Neutrophil-to-lymphocyte ratio (N/L) levels from admission to the seventh day post-ictus in the study population. Data are presented as a median and interquartile range of 25–75. The *p*-value was calculated using the Friedman test.

The median CRP and NLr on admission were 3.6 mg/L (IQR: 1.6–9.6) and 6.67 (IQR: 3.40–12.21), respectively (Supplementary Table S1). There was an increase over time in the daily median CRP levels (Figure 1A, *p* = 0.001), while NLr remained elevated but stable over time (Figure 1B, *p* = 0.57).

### 3.3 Neurological outcome

**Figure 2:**
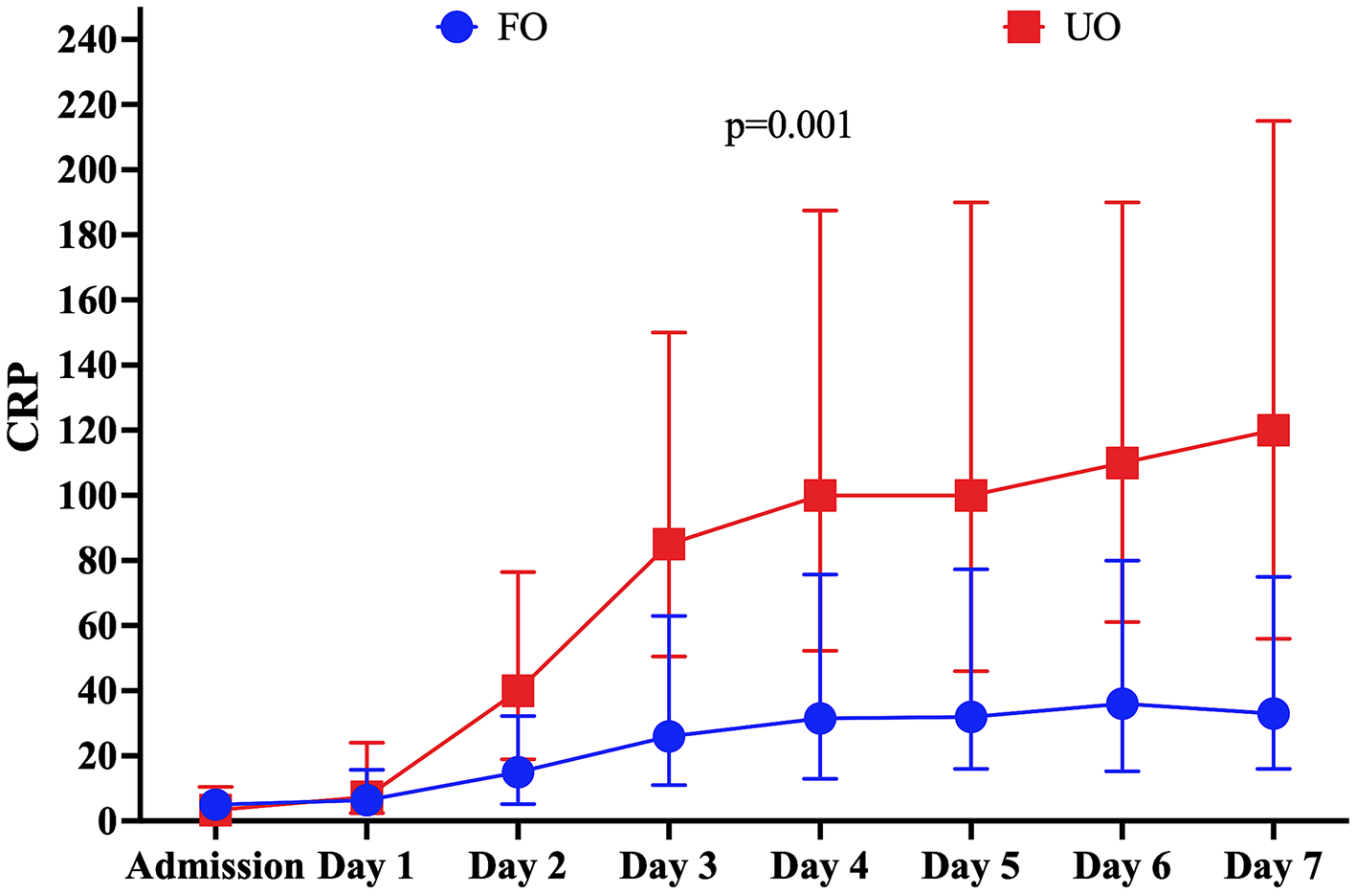
C-reactive protein (CRP) levels from admission to the seventh day post-ictus in the study population according to the neurological outcome at 3 months. Unfavorable outcomes (UOs) were defined as a Glasgow Outcome Scale score of 1–3 (death, vegetative state, and severe disability). Data are presented as a median and interquartile range of 25–75%. The *p*-value corresponds to the statistical significance of the interaction time and group in a mixed model.

Patients who had UOs were older and had a lower GCS on admission than patients with FOs. Patients with UOs also had a higher rate of neurological complications and infection than those with FOs (Table 1). The highest CRP value was significantly higher in the UO group [130.0 (56.3–220.0) mg/L vs. 45.0 (20.0–100.0) mg/L; *p* = 0.001]; although CRP progressively increased over time in both groups, CRP values were consistently higher from days 2 to 7 in patients with UOs than in patients with FOs (Supplementary Table S1 and Figure 2). In a multivariable logistic regression model, the highest CRP value (OR: 1.005; 95% CI: 1.0002–1.007) was independently associated with UOs when adjusted for confounders (Table 2).

**Table 2:** Logistic regression models to assess the association between the highest C-reactive protein level and the highest neutrophil-to-lymphocyte ratio (NLr) in the first 7 days after ictus and unfavorable neurological outcomes at 3 months.

Variables	Univariate analysis OR (95% CI)	Multivariable analysis OR (95% CI)	Variables	Univariate analysis OR (95% CI)	Multivariable analysis OR (95% CI)
Highest CRP	1.007 (1.005–1.009)	1.005 (1.002–1.007)	Highest NLr	1.05 (1.03–1.07)	1.03 (1.001–1.05)
Age	1.04 (1.03–1.06)	1.07 (1.05–1.09)	Age	1.04 (1.03–1.06)	1.07 (1.04–1.09)
WFNS 4–5	9.95 (6.71–14.75)	4.51 (2.64–7.71)	WFNS score of 4–5	9.95 (6.71–14.75)	5.58 (3.33–9.38)
Rebleeding	6.87 (2.82–16.74)	10.18 (2.92–35.54)	Rebleeding	6.87 (2.82–16.74)	9.30 (2.57–33.67)
DCI	4.52 (2.96–6.88)	5.54 (3.10–9.89)	DCI	4.52 (2.96–6.88)	5.40 (3.05–9.57)
ICHT	12.55 (8.27–19.04)	6.69 (3.90–11.49)	ICHT	12.55 (8.27–19.04)	7.29 (4.27–12.43)
Nimodipine	0.15 (0.08–0.28)	0.10 (0.04–0.22)	Nimodipine	0.15 (0.08–0.28)	0.12 (0.05–0.28)

An unfavorable outcome was considered as a Glasgow Outcome Scale score of 1–3 (death, vegetative state, and severe disability). CRP, C-reactive protein; NLr, neutrophil-to-lymphocyte ratio; WFNS, World Federation of Neurological Surgeons; DCI, delayed cerebral ischemia; ICHT, intracranial hypertension.

**Figure 3:**
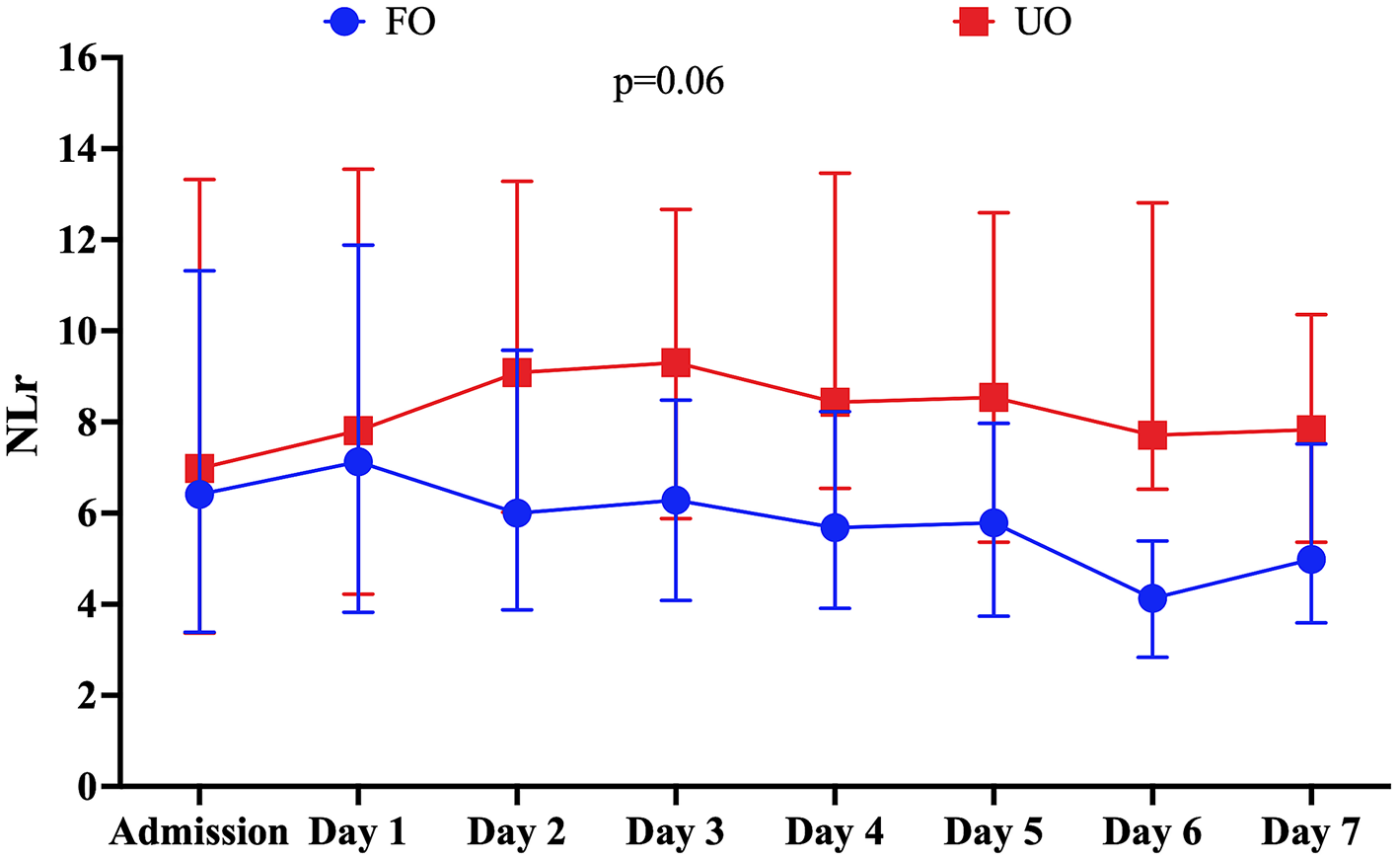
Neutrophil-to-lymphocyte ratio (N/L) levels from admission to the seventh day post-ictus in the study population according to the neurological outcome at 3 months. Unfavorable outcomes (UOs) were defined as a Glasgow Outcome Scale score of 1–3 (death, vegetative state, and severe disability). Data are presented as a median and interquartile range of 25–75%. The *p*-value corresponds to the statistical significance of the interaction time and group in a mixed model.

Similarly, the highest NLr value was significantly higher in the UO group [12.50 (8.28–17.65) vs. 9.01 (IQR: 5.38–13.69); *p* = 0.001]. Moreover, NLr values remained stable over time among patients with UOs, while they decreased in patients with FOs (Supplementary Table S1 and Figure 3). In a multivariable logistic regression model, the highest NLr value (OR: 1.025; 95% CI: 1.001–1.050) was independently associated with UOs when adjusted for confounders (Table 2).

When including both the highest CRP and the highest NLr in the same model, only CRP (OR: 1.005; 95% CI: 1.002–1.007) remained independently associated with unfavorable outcomes at 3 months (Table 3).

**Table 3:** Logistic regression model to assess the association between the highest C-reactive protein level and the highest neutrophil-to-lymphocyte ratio (NLr) in the first 7 days after ictus and unfavorable neurological outcomes at 3 months in the same model.

Variables	Multivariable analysis OR (CI 95%)
Highest CRP	1.005 (1.002–1.007)
Highest NLr	1.02 (0.99–1.04)
Age	1.07 (1.05–1.09)
WFNS score of 4–5	4.50 (2.64–7.70)
Rebleeding	10.24 (2.93–35.81)
DCI	5.38 (3.01–9.63)
Intracranial hypertension	6.73 (3.92–11.54)
Nimodipine	0.09 (0.40–0.21)

An unfavorable outcome was considered as a Glasgow Outcome Scale score of 1–3 (death, vegetative state, and severe disability). CRP, C-reactive protein; NLr, neutrophil-to-lymphocyte ratio; WFNS, World Federation of Neurological Surgeons; DCI, delayed cerebral ischemia.

### 3.4 Infections

**Figure 4:**
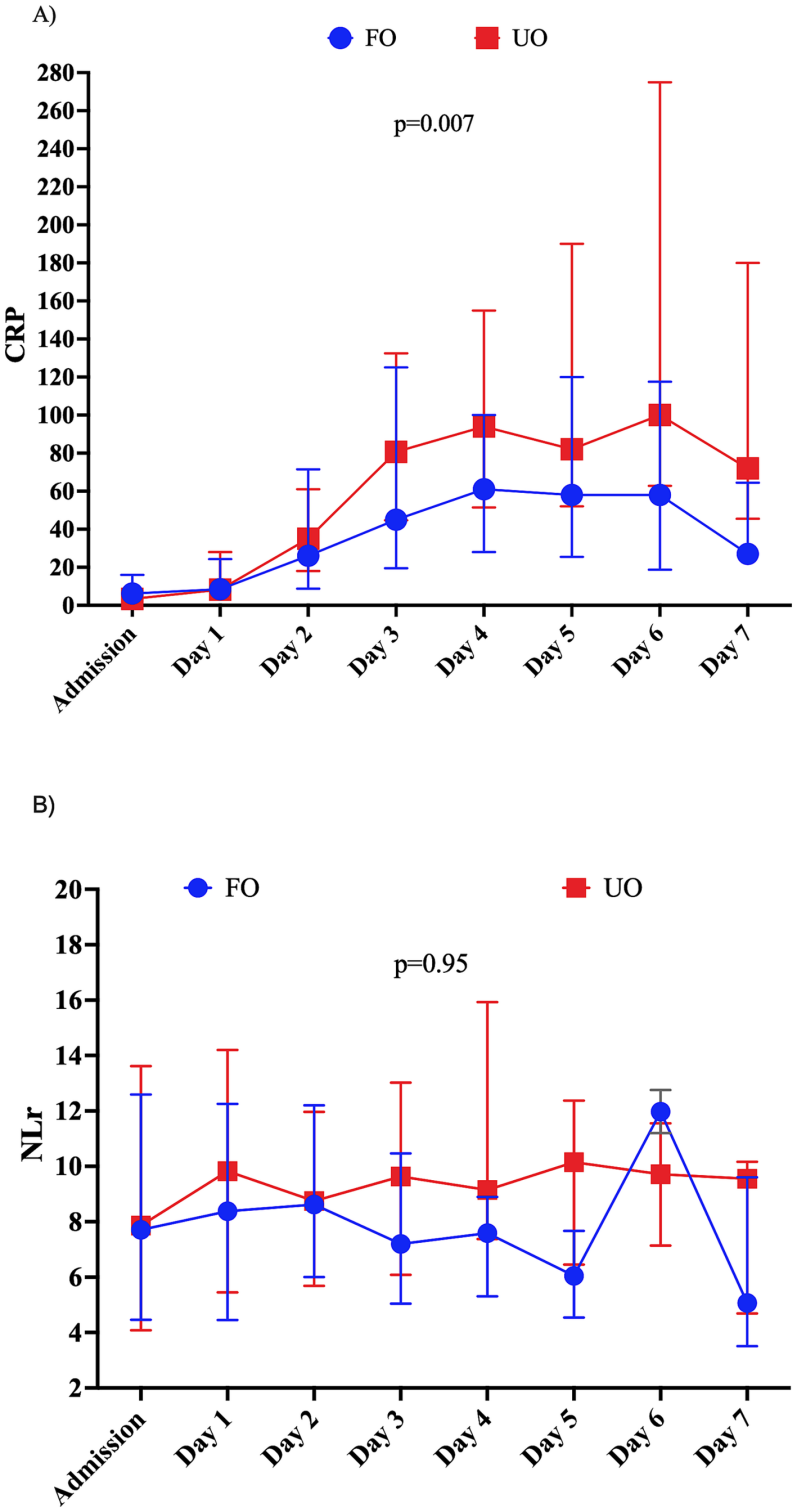
Evolution of **(A)** C-reactive protein (CRP) levels and **(B)** neutrophil-to-lymphocyte ratio (NLR) over time according to the development of infection in the first 7 days post-ictus. The *p*-value represents the statistical significance of the interaction between infection and CRP levels and between infection and NLR. CRP values are expressed in mg/L.

A total of 149 patients (27.2%) acquired infection during their ICU stay. The most common site of infection was pneumonia (80/149, 53.7%), followed by urinary tract infection (42/149, 28.2%) and bloodstream infection (20/149, 13.4%). Time from admission to infection was 5 days (IQR: 3–7). Patients with infections more frequently had a poor clinical grade on admission, had a higher prevalence of neurological complications, such as DCI, and had a higher frequency of UOs than patients without infection (Supplementary Table S2). Patients who developed infection had higher CRP (*p* = 0.001, Figure 4A) and NLr (*p* = 0.001, Figure 4B) values than those without infection.

**Figure 5:**
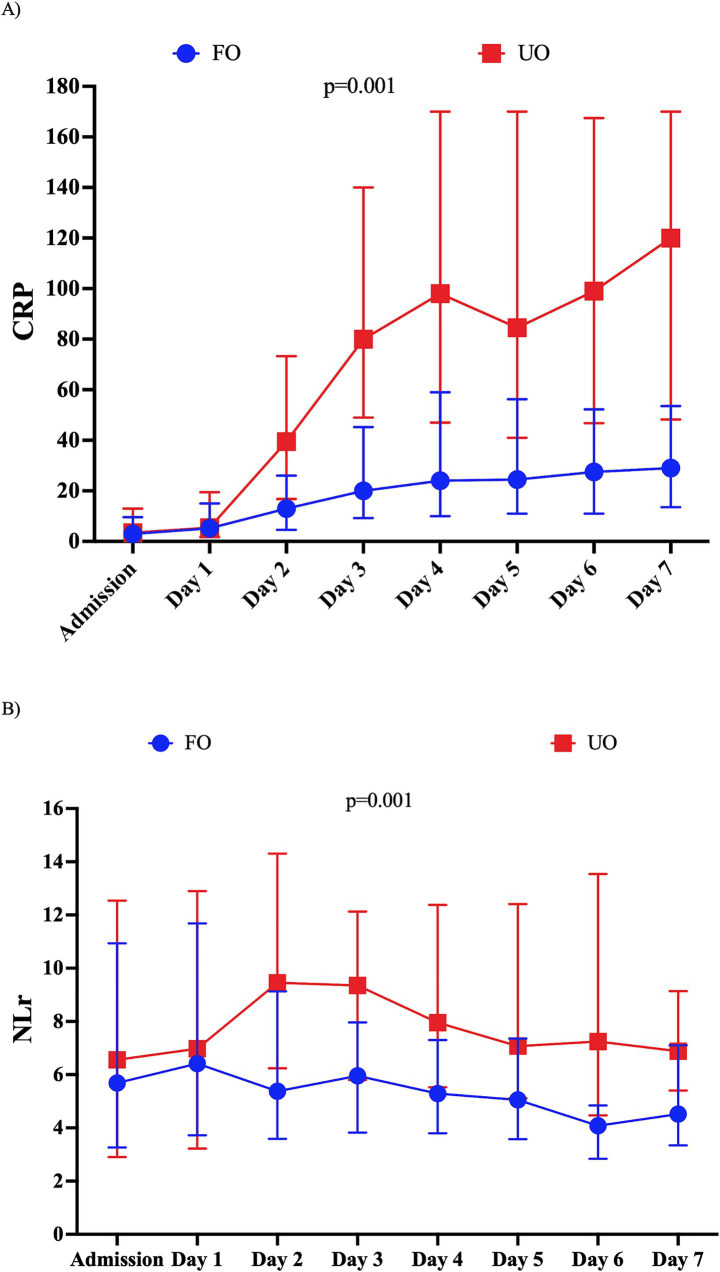
Evolution of **(A)** C-reactive protein (CRP) levels and **(B)** neutrophil-to-lymphocyte ratio (NLR) over time according to the neurological outcome at 3 months in patients who developed infection in the first 7 days post-ictus. CRP values are expressed in mg/L.

Considering only patients with infection, CRP was consistently higher from days 3 to 7 in patients with UOs compared with those with FOs (Figure 5A). However, in these patients, the highest CRP (OR: 1.01, 95% CI: 1.00–1.01) was not independently associated with UOs (Supplementary Table S3) when adjusted for confounders. Additionally, NLr remained statistically similar over time in patients with UOs and those with FOs (Figure 5B); in an adjusted logistic regression model, NLr (OR: 1.02; 95% CI: 0.99–1.06) was not independently associated with UOs (Supplementary Table S3).

**Figure 6:**
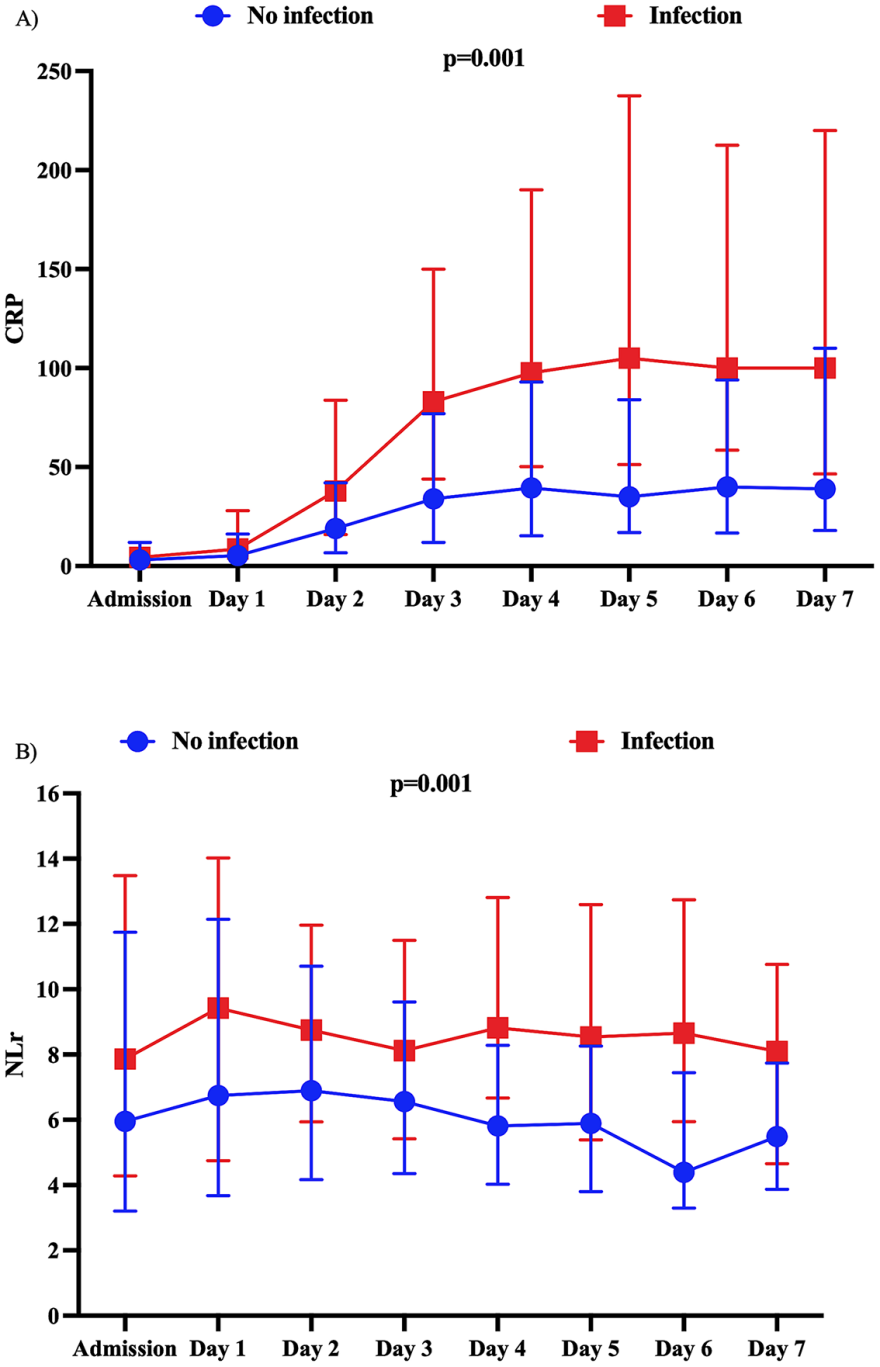
Evolution of **(A)** C-reactive protein (CRP) levels and **(B)** neutrophil-to-lymphocyte ratio (NLR) over time according to the neurological outcome at 3 months in patients without infection in the first 7 days post-ictus. CRP values are expressed in mg/L.

In patients without infection (Figures 6A,B), the highest CRP values (OR: 1.006; 95% CI: 1.001–1.010), but not the highest NLr values (OR: 1.02; 95% CI: 0.99–1.05), were significantly associated with UOs when adjusted for confounders (Supplementary Table S4).

### 3.5 Delayed cerebral ischemia

Patients who developed DCI had higher clinical and neurological severity on admission and a higher prevalence of hydrocephalus, intracranial hypertension, angiographic vasospasm, and infection than others (Supplementary Table S5). Supplementary Figure S1 shows that CRP (panel A, *p* = 0.001) and NLr (panel B, *p* = 0.001) daily values were higher in the first 7 days of ICU stay in patients with DCI than those without DCI.

The highest NLr (sHR: 1.02; 95% CI: 1.01–1.03) in the first 7 days, but not CRP (sHR: 1.00; 95% CI: 1.00–1.01), was independently associated with the development of DCI (Supplementary Table S6) in a competing risk analysis adjusted for age, sex, history of systemic hypertension, an mFisher score of 3–4, a WFNS score of 4–5, and angiographic vasospasm, with the competing event defined as ICU death. This association remained significant in a competing risk analysis considering only patients with an infection (highest NLr/sHR: 1.03; 95% CI: 1.01–1.05) but not in patients without infection (highest NLr/sHR: 1.01; 95% CI: 0.98–1.03), as shown in Supplementary Tables S7, S8.

Patients with infection who had developed DCI had higher CRP (*p* = 0.008) and NLr (*p* = 0.001) levels over time than those who had infection but did not have DCI (Supplementary Figure S2). Conversely, patients without infection who had developed DCI had comparable daily levels of CRP and NLr over time (Supplementary Figure S3) to patients without infection who did not develop DCI.

## 4 Discussion

In this retrospective single-center study on aSAH patients, elevated biomarkers of systemic inflammation, such as CRP and NLr, over the first week after bleeding were associated with a higher chance of unfavorable neurological outcomes at 3 months. Moreover, the highest NLr level was also associated with the development of DCI.

A systematic review and meta-analysis that analyzed 12 studies (20, 52–62), with a total of 4,840 SAH patients, revealed an independent association (OR: 1.31; 95% CI: 1.14–1.49) between admission NLr and poor neurological outcomes at 3 months (21). Of note, only 5 studies (52, 55, 57, 58, 62, and) of the 12 studies reported this association. In our study, we showed that the highest NLr in the first 7 days was associated with outcomes. Interestingly, this association was not significant in the univariate analysis, but only after considering the impact of clinical grade on admission assessed by the WFNS score, did we see this significant association between NLr and neurological outcomes. As previously described, patients with poor clinical grade can mount a more robust inflammatory response than patients with good grade after the initial hemorrhage (13).

Additionally, patients with favorable and unfavorable neurological outcomes had similar NLr in the first 24 h; however, from days 2 to 7, NLr remained elevated in patients with UOs, while in patients with FOs, NLr tended to decrease. This suggests that a “dose-effect” association with outcome, in which not only the peak of NLr is important, but also the time spent with elevated NLr.

Delayed cerebral ischemia has a complex pathophysiology, which includes neuroinflammation. In fact, leukocytosis and lymphopenia, represented by NLr, can promote microcirculatory dysfunction and contribute to microthrombi formation and cortical ischemia, all of which are processes involved in the development of DCI (63). Interestingly, the association between NLr and DCI may also reflect the role of neutrophil extracellular traps (NETs) in the pathology of DCI (64). NETs are a downstream neutrophil-mediated immune mechanism that can cause vascular endothelial injury and thrombosis (65). Previous studies have consistently identified NET biomarkers in the serum of aneurysmal subarachnoid hemorrhage patients (66, 67) and have shown an association between these biomarkers and delayed cerebral ischemia (68). In this setting, an association between NLr in the first 24 h after bleeding and the development of DCI has been consistently reported (20, 56, 58, 62, 69, 70) and was further confirmed in our study. This highlights the interplay between neuroinflammation and systemic inflammation.

Different from previous studies (15, 71–73), we did not observe an association between the highest CRP in the first 7 days after SAH and delayed cerebral ischemia. Conversely, we showed that elevated CRP was also associated with poor outcomes after SAH. Similarly, Lee et al. (74) also reported an association between the highest CRP in the first 7 days post-bleeding and poor neurological outcomes, while other studies showed an association between an early increase in CRP during early brain injury and poor outcomes, often independently of the development of DCI (16, 23, 28, 29, 31, 75–77).

Interestingly, when included in the same model, only CRP remained independently associated with poor outcomes, while NLr did not. Other studies (24, 78) have focused on the ratio between CRP and lymphocytes (CRL) and demonstrated an association between higher levels of CRL, DCI, and poor outcomes, possibly representing both increased inflammatory response (high CRP) and stroke-related immunodepression (low lymphocyte count) (79).

Importantly, NLr is easily obtained by performing a blood cell count, without any additional costs and with minimal risk of harm to patients. Similarly, CRP measurements are cheap, readily available, consistent, and reproducible in the majority of countries (74). Despite the low-quality level of evidence, as all studies are observational and conducted mostly in a single center, NLr and CRP may be useful as additional biomarkers for the identification of patients at risk of DCI and to help with neuro-prognostication in both low-and middle- and high-income countries (77).

Of note, infections, especially pneumonia, are a common medical complication (80) that significantly impacts the outcome of aSAH patients (81). In clinical practice, CRP and neutrophil count are biomarkers used to aid the diagnosis of infection and to monitor response to treatment (82). In our study, 27% of patients acquired an infection in the first week of hospitalization. In fact, infection is an important confounder when interpreting our results. In patients who acquired an infection, elevated levels of CRP and NLr are likely due to an inflammatory response to fight the infection rather than a systemic inflammatory response to the primary subarachnoid bleed. Additionally, the association between CRP/NLr and the outcome in infected patients may indirectly reflect the impact of infectious complications on the outcome, as previously described (83).

Our study has some limitations. As a retrospective study, data collection may be subject to biases related to clinical recording and is limited by missing data, and we cannot exclude the presence of unaccounted confounders that may have influenced our results. As a single-center study, our results may be applicable only to cohorts with characteristics similar to ours, and local practices may have impacted our results. Moreover, due to a lack of available data regarding long-term neurological assessment, we limited our investigation to short-term outcomes. Additionally, we chose to exclude patients with imminent death due to catastrophic injuries by excluding those who stayed less than 24 h in the ICU. However, by doing so, we failed to study the impact of inflammatory biomarkers on the mechanism of early death. We were also unable to assess the presence of inflammatory biomarkers in the central nervous system. In our center, no standardized guidelines on the collection of CRP exist, although blood cell counts are usually performed daily in the acute phase; the decision of whether, when, and how to monitor these biomarkers was made by the healthcare team, which may have impacted our results. An ideal biomarker would be monitored continuously and in real time. This, unfortunately, is not available for these biomarkers in a retrospective setting. Additionally, despite adjustments in the multivariable analysis for the most commonly described factors associated with the outcome of SAH patients, confounders not accounted for may have impacted our results. Propensity score matching may be a valid method to better address this bias, and future studies should consider it.

## 5 Conclusion

In this SAH cohort, commonly used biomarkers of systemic inflammation, such as C-reactive protein and neutrophil-to-lymphocyte ratio, were frequently elevated in the early phase after the initial bleed and were associated with the development of DCI and an increased risk of unfavorable outcomes.

## Data Availability

The original contributions presented in the study are included in the article/Supplementary material, further inquiries can be directed to the corresponding author.
