# Supplementary material for: Association between systemic inflammation biomarkers and outcomes in patients with aneurysmal subarachnoid hemorrhage
Source: Front Neurol. 2026 Mar 17;17:1749871. doi: 10.3389/fneur.2026.1749871 (PMC13035489; doi:10.3389/fneur.2026.1749871)
Supplement: Supplementary file 1 [file Data_Sheet_1.PDF]

Supplementary Material

1 Supplementary Tables

Supplemental table S1:C-reactive protein and Neutrophil lymphocyte ratio in the first 7 days post ictus according to neurological ouctome at 3 months. Favorable outcome (FO) was defined as a Glasgow outcome scale (GOS) of 4-5 and unfavorable ouctome was defined as a GOS 1-3.

	All (n=547)	FO (n=297)	UO (n=250)	p -value
**C-reactive protein (mg/L), median (IQR 25%-75%)**				
**Admission**	3.6 (1.60; 9.6)	5.0 (1.7; 9.8)	3.4 (1.5; 9.0)	0.573
**Day 1**	6.9 (2.3; 19.0)	6.5 (2.1; 15.5)	7.4 (2.4; 24.0)	0.232
**Day 2**	23.0 (8.9; 52.0)	15.0 (5.2; 32.0)	40.0 (19.0; 76.0)	0.001
**Day 3**	47.5 (18.0; 100.0)	26.0 (11.0; 62.5)	85.0 (51.0; 150.0)	0.001
**Day 4**	55.0 (21.5; 120.0)	31. 5 (13.0; 75.5)	100.0 (52.5; 185.0)	0.001
**Day 5**	58.0 (23.5; 130.0)	32.0 (16.0; 77.0)	100.0 (46.0; 190.0)	0.001
**Day 6**	61.0 (25.0; 150.0)	36.0 (15.5; 80.0)	110.0 (61.3; 190.0)	0.001
**Day 7**	60.0 (23.0; 150.0)	33.0 (17.0; 74.5)	120.0 (56.0; 210.0)	0.001
**Highest CRP**	73.0 (24.0; 170.0)	45.0 (20.0; 100.0)	130.0 (56.0; 220.0)	0.001
**Neutrophil lymphocyte ratio, median (IQR 25%-75%)**				
**Admission**	6.67 (3.40- 12.21)	6.41 (3.39-11.30)	6.98 (3.23-13.11)	0.46
**Day 1**	6.70 (3.39; 12.21)	6.41 (3.39; 11.30)	6.98 (3.43; 13.11)	0.19
**Day 2**	7.81 (4.79; 11.33)	6.00 (3.90;9,57)	9.08 (6.03; 13.22)	0.001
**Day 3**	7.00 (4.58; 10.27)	6.28 (4.10; 8.44)	9.31 (5.89; 12.45)	0.001
**Day 4**	6.91 (4.71; 9.71)	5.68 (3.91; 8.21)	8.44 (6.60; 13.37)	0.001
**Day 5**	6.42 (4.04; 10.07)	5.79 (3.76; 7.95)	8.53 (5.40; 12.55)	0.001

**Day 6**	5.71 (3.96; 10.05)	4.13 (2.84; 5.35)	7.71 (6.54; 12.77)	0.001
**Day 7**	6.45 (4.17; 9.56)	4.99 (3.60; 7.50)	7.83 (5.40; 10.36)	0.001
**Highest NLr**	10.60 (6.69; 15.88)	9.01 (5.38; 13.69)	12.50 (8.28; 17.65)	0.001

FO: favorable outcome; UO: unfavorable outcome; CRP: c-reactive protein;NLr: neutrophil lymphocyte ratio.

Supplemental table S2 : Characteristics of the study population according to the development of infection in the first 7 days post ictus.

	**No infection (N=398)**	**Infection (n=149)**	**p -value**
**Age (years), median (IQR 25-75%)**	53 (45;62)	55 (46;64)	0.21
**Female gender, n (%)**	251 (63.1)	87 (58.4)	0.32
**APACHE II, median (IQR 25-75%)**	10 (7;17)	17 (11; 20)	0.001
**SOFA score, median (IQR 25-75%)**	2 (1;7)	6 (4; 9)	0.001
**GCS, median (IQR)**	14 (6;15)	7 (3; 14)	0.001
**mFisher 3-4, n (%)**	347 (88.7)	147 (99.3)	0.001
**WFNS 4-5, n (%)**	153 (38.4)	96 (64.4)	0.001
**Nimodipine (prophylaxis)**	339 (85.2)	136 (91.3)	0.07
**Aneurysm treatment, n (%)**			0.01
**Endovascular treatment of aneurysm**	358 (89.9)	122 (81.9)	
**Surgical treatment of aneurysm**	40 (10.1)	27 (18.1)	
**Comorbidities, n (%)**			
**Systemic arterial hypertension**	171 (43.0)	60 (40.5)	0.63
**Diabetes Mellitus**	30 (7.6)	15 (10.1)	0.38
**Heart disease**	48 (12.1)	15 (10.1)	0.65
**Previous Neurological Disease**	24 (6.0)	14 (9.5)	0.99
**CKD**	7 (1.8)	2 (1.4)	0.99
**Asthma/COPD**	30 (7.5)	17 (11.5)	0.17

**Cancer**	20 (5.0)	6 (4.1)	0.64
**Cirrhosis**	5 (1.3)	2 (1.4)	0.99
**ICU management, n (%)**			
**Vasopressor use**	192 (48.2)	113 (75.8)	0.001
**Inotrope use**	53 (13.3)	36 (24.2)	0.004
**Mechanical Ventilation**	190 (47.7)	126 (84.6)	0.001
**Complications, n (%)**			
**Seizures**	70 (17.6)	58 (38.9)	0.001
**Rebleeding**	29 (7.3)	8 (5.4)	0.57
**Hydrocephalus**	112 (28.4)	72 (48.3)	0.001
**Delayed cerebral ischemia**	80 (20.1)	60 (40.5)	0.001
**Intracranial hypertension**	137 (34.5)	79 (54.1)	0.001
**C-reactive protein (mg/L), median (IQR 25%-75%)**			
**Admission**	3.2 (1.4;12.0)	4.4 (1.7; 7.1)	0.63
**Day 1**	5.4 (1.8;16)	8.6 (3.6; 28.0)	0.001
**Day 2**	19.0 (7.0;41.0)	38.0 (16.0; 83.0)	0.001
**Day 3**	34 (12.0-76.0)	83.0 (44.0; 150.0)	0.001
**Day 4**	39.5 (15.5;93)	97.7 (50.5; 190.0)	0.001
**Day 5**	35.0 (17.0;84.0)	105.0 (51.5; 235.0)	0.001
**Day 6**	40.0 (17.0;94.0)	100.0 (59.0; 210.0)	0.001
**Day 7**	39.0 (18.0; 105.0)	60.0 (23.0; 150.0)	0.001

**Highest CRP**	49.5 (18.0;110.0)	190.0 (99.0; 280.0)	0.001
**Neutrophil lymphocyte ratio, median (IQR 25%-75%)**			
**Admission**	5.96 (3.20;11.75)	7.86 (4.34- 13.48)	0.03
**Day 1**	6.74 (3.68;12.14)	9.43 (4.95; 14.01)	0.006
**Day 2**	6.90 (4.18;10.68)	8.75 (6.00; 11.96)	0.001
**Day 3**	6.56 (4.35;9.55)	8.12 (5.47; 11.47)	0.002
**Day 4**	5.81 (4.06;8.26)	8.81 (6.73; 12.25)	0.001
**Day 5**	5.89 (3.80;8.23)	8.54 (5.43; 12.55)	0.001
**Day 6**	4.39 (6.16;7.44)	8.65 (6.16; 12.72)	0.001
**Day 7**	5.49 (3.88; 7.74)	6.45 (4.17; 9.56)	0.001
**Highest NLr**	9.29 (5.51;14.60	13.46 (10.00; 19.13)	0.001
**Outcomes**			
**ICU LOS – days median (IQR 25% - 75%)**	4 (2;11)	15 (10; 21)	0.001
**Hospital LOS– days median (IQR 25% - 75%)**	16 (6; 26)	23 (12; 46)	0.001
**Unfavourable neurological outcome at 3 months, n (%)**	156 (39.2)	94 (63.1)	0.001

APACHE: Acute Physiology and Chronic Health Evaluation; SOFA: sequential Organ Failure Assessment; GCS: Glasgow coma scale; ICU: Intensive care unit; MV: mechanical ventilation; WFNS: world federation of neurosurgical societies; DM: diabetes mellitus; ND: neurological disease; CRF: chronic renal failure; COPD: chronic obstructive pulmonary disease; RRT: renal replacement therapy; ECMO: extracorporeal membrane oxygenation; EVD: external ventricular drain; ICP: intracranial pressure; cEEG: continuous electroencephalogram; DCI: delayed cerebral ischemia; ICHT: intracranial hypertension; IA: intra-arterial; BSI: blood stream infection; CNS: central nervous system; LOS: length of stay.

Supplemental Table S3: Univariate and multivariate logistic regression to assess the association of the highest CRP and NLr in the first 07 days post ictus and neurological outcome at 3 months in patients who developed infection during the first week after ictus.

Variables	Univariate analysis<br>OR (95% CI	Multivariable analysis<br>OR (CI 95%)	Variables	Univariate analysis<br>OR (95% CI	Multivariable analysis<br>OR (CI 95%)
**Highest CRP**	1.003 (1.000-1.006)	1.01 (1.00-1.007)	**Highest NLr**	1.02 (0.99-1.05)	1.02 (0.99-1.06)
**Age**	1.03 (1.002-1.06)	1.07 (1.03-1.11)	**Age**	1.03 (1.002-1.06)	1.06 (1.03-1.10)
**WFNS 4-5**	2.25 (1.26-5.06)	1.86 (0.77-4.47)	**WFNS 4-5**	2.25 (1.26-5.06)	2.41 (1.02-5.72)
**Rebleeding**	4.35 (0.52-36.29)	4.06 (0.27-60.29)	**Rebleeding**	4.35 (0.52-36.29)	4.06 (0.27-60.29)
**DCI**	5.23 (2.36-11.61)	11.71 (4.31-31.79)	**DCI**	5.23 (2.36-11.61)	10.38 (4.05-28.99)
**ICHT**	2.37 (1.19-4.71)	3.43 (1.39-8.43)	**ICHT**	2.37 (1.19-4.71)	3.76 (1.52-9.29)
**Nimodipine**	0.26 (0.06-1.20)	0.10 (0.02-0.60)	**Nimodipine**	0.26 (0.06-1.20)	0.08 (0.01-0.50)

CRP: C-reactive protein; NLr: neutrophil/lymphocyte ratio;WFNS: World federation of neurological surgeons; DCI: delayed cerebral ischemia; OR: odds ratio; CI: confidence interval; ICHT = intracranial hypertension.

Supplemental Table S4: Univariate and multivariate logistic regression to assess the association of the highest CRP and NLr in the first 07 days post ictus and neurological outcome at 3 months in patients who did not developed infection during the first week after ictus.

Variables	Univariate analysis<br>OR (95% CI	Multivariable analysis<br>OR (CI 95%)	Variables	Univariate analysis<br>OR (95% CI	Multivariable analysis<br>OR (CI 95%)
**Highest CRP**	1.008 (1.005-1.011)	1.006 (1.001-1.010)	**Highest NLr**	1.05 (1.02-1.08)	1.02 (0.99-1.05)
**Age**	1.04 (1.03-1.06)	1.07 (1.04-1.10)	**Age**	1.04 (1.03-1.06)	1.07 (1.04-1.10)
**WFNS 4-5**	15.93 (9.64-26.32)	7.81 (3.88-15.70)	**WFNS 4-5**	15.93 (9.64-26.32)	8.86 (4.46-17.63)
**Rebleeding**	8.68 (3.24-23.30)	13.85 (3.30-58.15)	**Rebleeding**	8.68 (3.24-23.30)	14.62 (3.39-63.03)
**DCI**	3.60 (2.16-6.01)	2.61 (1.18-5.79)	**DCI**	3.60 (2.16-6.01)	2.66 (1.22-5.79)
**ICHT**	27.14 (15.49-47.54)	14.13 (6.87-29.07)	**ICHT**	27.14 (15.49-47.54)	14.60 (7.13-29.86)
**Nimodipine**	0.52 (0.33-0.81)	0.32 (0.19-0.71)	**Nimodipine**	0.52 (0.33-0.81)	0.42 (0.20-0.89)

CRP: C-reactive protein; NLr: neutrophil/lymphocyte ratio;WFNS: World federation of neurological surgeons; DCI: delayed cerebral ischemia; OR: odds ratio; CI: confidence interval; ICHT = intracranial hypertension

Supplemental Table S5 : Characteristics of the study population according to the development of delayed cerebral ischemia (DCI).

	No DCI (N=407)	DCI (n=140)	p -value
**Age (years), median (IQR 25-75%)**	55 (46;63)	52 (45;62)	0.24
**Female gender, n (%)**	249 (61.2)	89 (63.3)	0.69
**APACHE II, median (IQR 25-75%)**	11(7;18)	15 (10; 19)	0.001
**SOFA score, median (IQR 25-75%)**	3 (1;7)	6 (2; 9)	0.001
**GCS, median (IQR)**	14 (5;15)	10 (3; 14)	0.001
**mFisher 3-4, n (%)**	363 (91.0)	131 (93.6)	0.38
**WFNS 4-5, n (%)**	167 (41.0)	82 (58.6)	0.001
**Nimodipine (prophylaxis), n (%)**	343 (84.3)	132 (94.3)	0.002
**Aneurysm treatment, n (%)**			0.30
**Endovascular treatment of aneurysm**	361 (88.7)	119 (85.0)	
**Surgical treatment of aneurysm**	46 (11.3)	21 (15.0)	
**Comorbidities, n (%)**			
**Systemic arterial hypertension**	167 (41.1)	64 (45.7)	0.37
**Diabetes Mellitus**	30 (7.4)	15 (10.7)	0.22
**Heart disease**	48 (11.8)	15 (10.7)	0.88
**Previous Neurological Disease**	31 (7.6)	7 (5.0)	0.34
**CKD**	7 (1.7)	2 (1.4)	0.99
**Asthma/COPD**	32 (7.9)	15 (10.7)	0.30

**Cancer**	21 (5.2)	5 (3.6)	0.65
**Cirrhosis**	3 (0.7)	4 (2.9)	0.08
**ICU management, n (%)**			
**Vasopressor use**	184 (45.2)	121 (86.4)	0.001
**Inotrope use**	48 (11.8)	41 (29.3)	0.001
**Mechanical Ventilation**	204 (50.1)	112 (80.0)	0.001
**Complications, n (%)**			
**Seizures**	81 (19.9)	47 (33.6)	0.002
**Rebleeding**	24 (5.9)	13 (9.3)	0.18
**Hydrocephalus**	119 (29.2)	66 (47.1)	0.001
**Vasospasm**	105 (25.8)	113 (81.3)	0.001
**Intracranial hypertension**	136 (33.4)	80 (58.0)	0.001
**C-reactive protein (mg/L), median (IQR 25%-75%)**			
**Admission**	3.7 (1.4;9.6)	4.2 (1.7; 11.0)	0.43
**Day 1**	5.8 (2.0;16.0)	8.1 (3.1; 26.0)	0.007
**Day 2**	21.0 (7.2;45.0)	35.0 (15.0; 62.0)	0.001
**Day 3**	39.0 (13.0-86.5)	72.0 (35.0; 140.0)	0.001
**Highest CRP**	31.0 (10.0;77.0)	64.0 (30.0; 140.0)	0.001
**Neutrophil lymphocyte ratio, median (IQR 25%-75%)**			
**Admission**	6.44 (3.19;11.30)	7.10 (4.27- 13.60)	0.65
**Day 1**	7.43 (3.72;12.21)	7.81 (4.53; 13.84)	0.18
**Day 2**	7.43 (4.54;10.65)	8.52 (5.64; 13.46)	0.05

**Day 3**	6.68 (4.34;9.60)	8.28 (5.88; 12.67)	0.001
**Highest NLr**	8.66 (4.96;13.56)	11.88 (6.90; 16.85)	0.001
**Infection n (%)**	89 (21.9)	60 (42.9)	0.001
**Outcomes**			
**ICU LOS – days median (IQR 25% - 75%)**	5 (2;12)	15 (8; 22)	0.001
**Hospital LOS– days median (IQR 25% - 75%)**	17 (8; 26)	23 (12; 41)	0.001
**Unfavourable neurological outcome at 3 months, n (%)**	149 (36.6)	101 (72.1)	0.001

APACHE: Acute Physiology and Chronic Health Evaluation; SOFA: sequential Organ Failure Assessment; GCS: Glasgow coma scale; ICU: Intensive care unit; MV: mechanical ventilation; WFNS: world federation of neurosurgical societies; DM: diabetes mellitus; ND: neurological disease; CRF: chronic renal failure; COPD: chronic obstructive pulmonary disease; RRT: renal replacement therapy; ECMO: extracorporeal membrane oxygenation; EVD: external ventricular drain; ICP: intracranial pressure; cEEG: continuous electroencephalogram; DCI: delayed cerebral ischemia; ICHT: intracranial hypertension; IA: intra-arterial; BSI: blood stream infection; CNS: central nervous system; LOS: length of stay.

Supplemental Table S6: Multivariate competing risk analysis to assess the association of the highest CRP and NLr and the occurrence of delayed cerebral ischemia (DCI). All variables included in the model preceded the development of DCI. ICU Death was considered a competing risk.

Variables	Multivariable analysis<br>sHR (CI 95%)
**Highest CRP**	1.00 (1.00-1.01)
**Highest NLr**	1.02 (1.01-1.03)
**mFisher 3-4**	0.61 (0.31-1.20)
**WFNS 4-5**	1.26 (0.90-1.77)
**Vasospasm**	5.13 (3.36-7.83)
**Age**	1.00 (0.98-1.01)
**Female sex**	1.10 (0.81-1.50)
**History of Systemic hypertension**	1.23 (0.91-1.67)

CRP: C-reactive protein; NLr: neutrophil/lymphocyte ratio;WFNS: World federation of neurological surgeons; OR: odds ratio; CI: confidence interval.

Supplemental Table S7: Multivariate competing risk analysis to assess the association of the highest CRP and NLr and the occurrence of delayed cerebral ischemia (DCI) in patients with infection. All variables included in the model preceded the development of DCI. ICU Death was considered a competing risk.

Variables	Multivariable analysis<br>sHR (CI 95%)
**Highest CRP**	1.00 (0.99-1.01)
**Highest NLr**	1.03 (1.01-1.05)
**mFisher 3-4**	1.29 (1.02-1.64)

**WFNS 4-5**	1.09 (0.62-1.91)
**Vasospasm**	5.35 (2.56-11.15)
**Age**	0.98 (0.96-1.01)
**Female sex**	1.09 (0.63-1.89)
**History of Systemic hypertension**	1.46 (0.86-2.47)

CRP: C-reactive protein; NLr: neutrophil/lymphocyte ratio;WFNS: World federation of neurological surgeons; OR: odds ratio; CI: confidence interval.

Supplemental Table S8: Multivariate competing risk analysis to assess the association of the highest CRP and NLr and the occurrence of delayed cerebral ischemia (DCI) in patients without infection. All variables included in the model preceded the development of DCI. ICU Death was considered a competing risk.

**Variables**	**Multivariable analysis**<br>**sHR (CI 95%)**
**Highest CRP**	1.00 (0.99-1.00)
**Highest NLr**	1.01 (0.98-1.03)
**mFisher 3-4**	0.60 (0.30-1.19)
**WFNS 4-5**	1.36 (0.87-2.13)
**Vasospasm**	4.90 (2.92-8.20)
**Age**	1.01 (0.99-1.02)
**Female sex**	1.09 (0.73-1.62)
**History of Systemic hypertension**	1.10 (0.74-1.61)

2 Supplementary Figures

Supplementary Figure S1: Evolution of C-reactive protein (CRP- panel A) and neutrophil/lymphocyte ratio (N/L- panel B) levels over time according to the presence of delayed cerebral ischemia.

A)

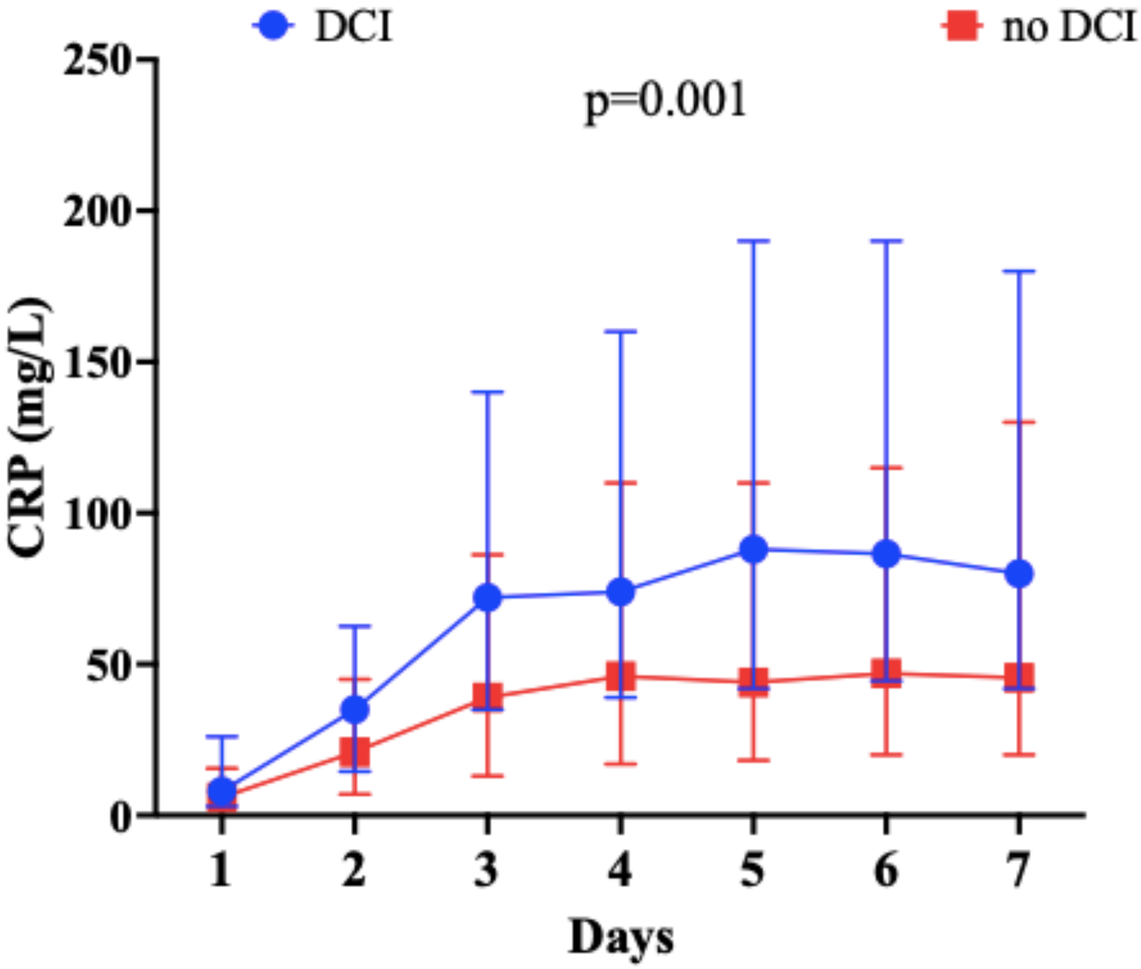

B)

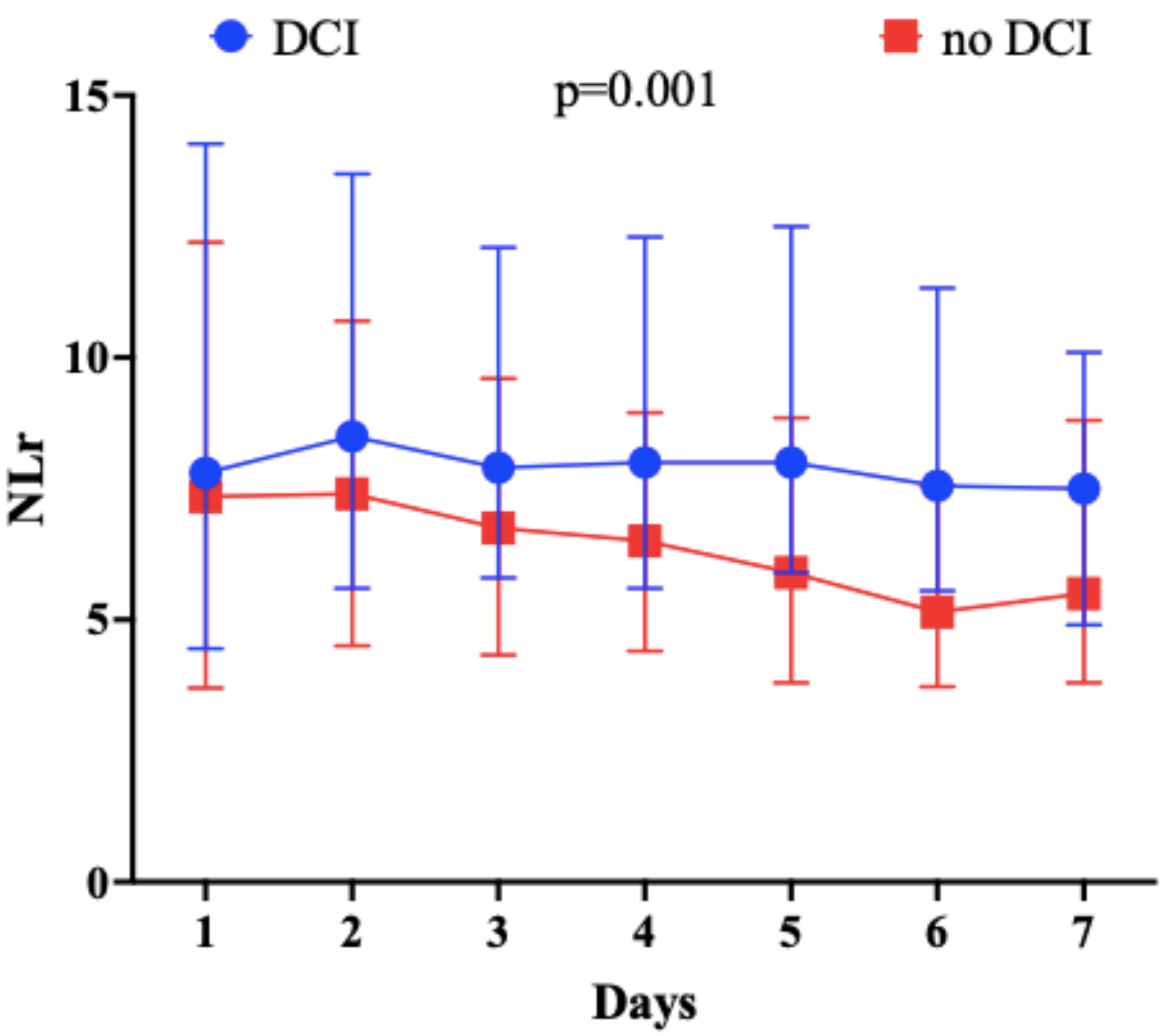

Supplementary Figure S2: Evolution of C-reactive protein (CRP- panel A) and neutrophil/lymphocyte ratio (N/L- panel B) levels over time according to the presence of delayed cerebral ischemia in patients who developed infection.

A)

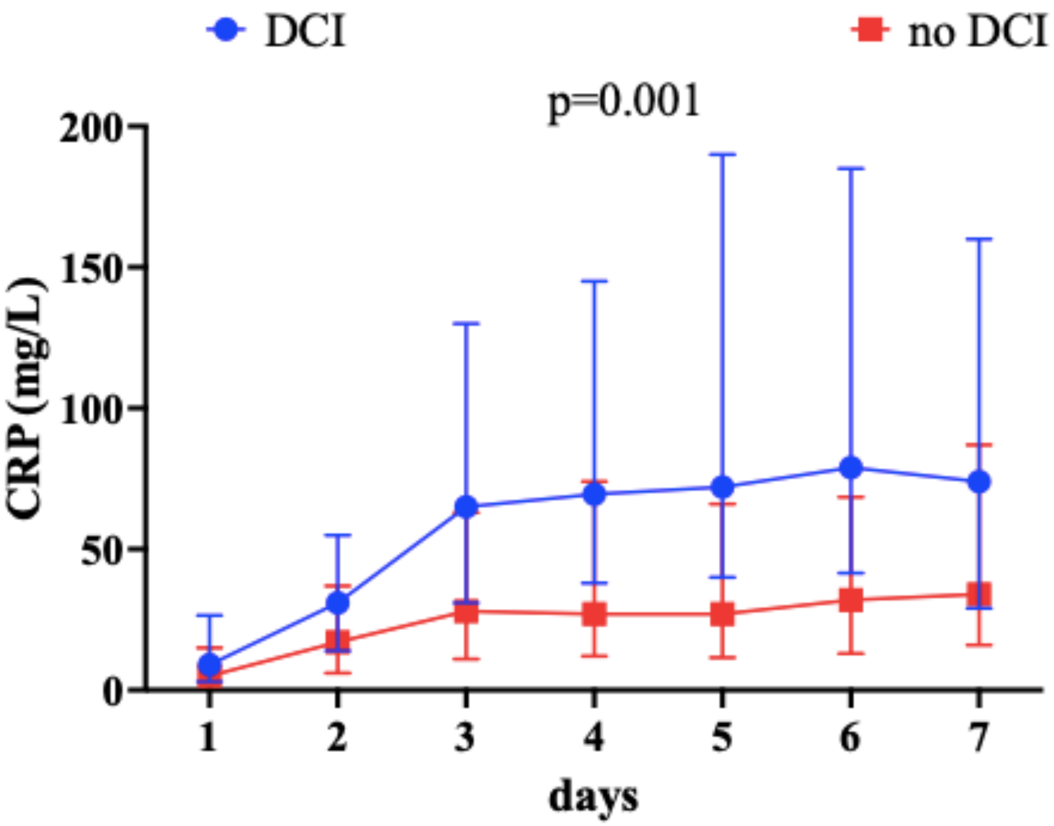

B)

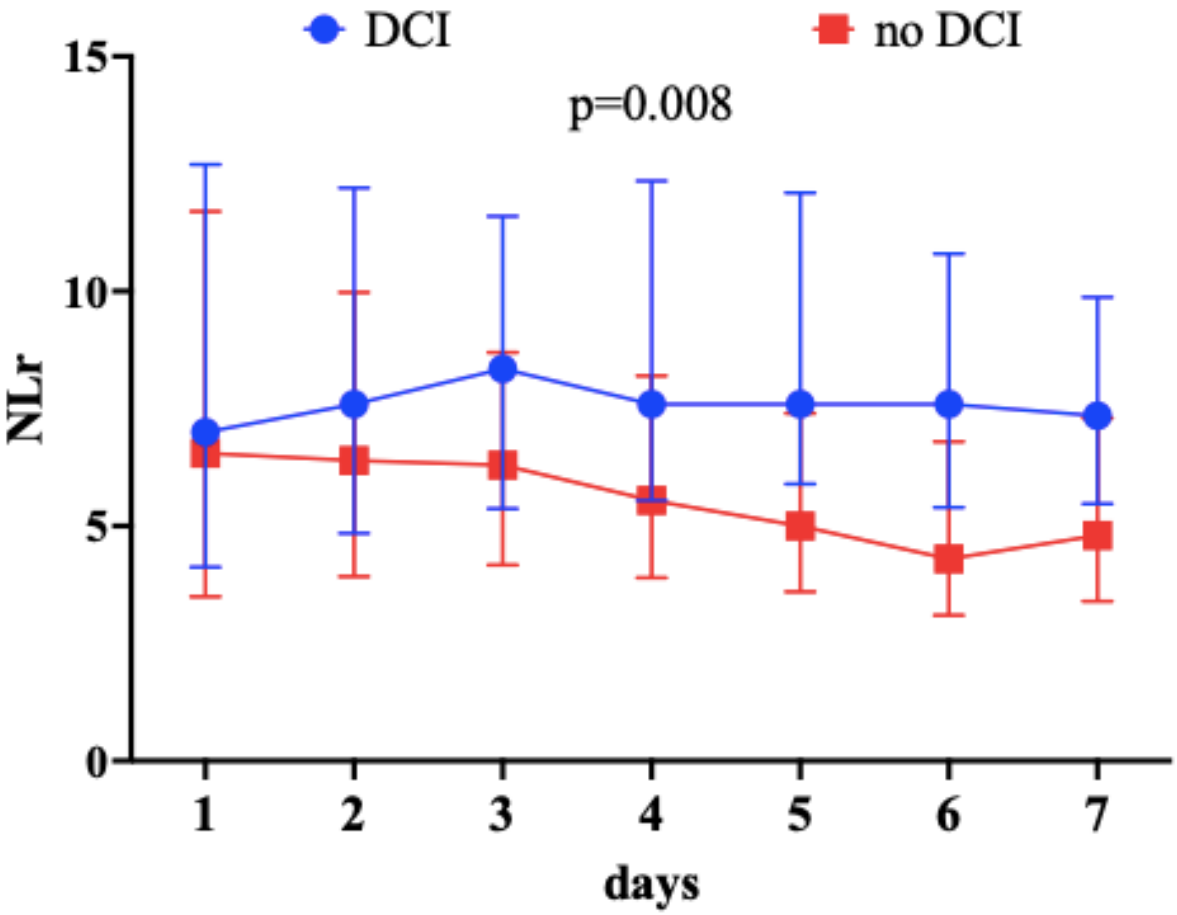

Supplemental Figure S3: Evolution of C-reactive protein (CRP- panel A) and neutrophil/lymphocyte ratio (N/L- panel B) levels over time according to the presence of delayed cerebral ischemia in patients who remained infection free.

A)

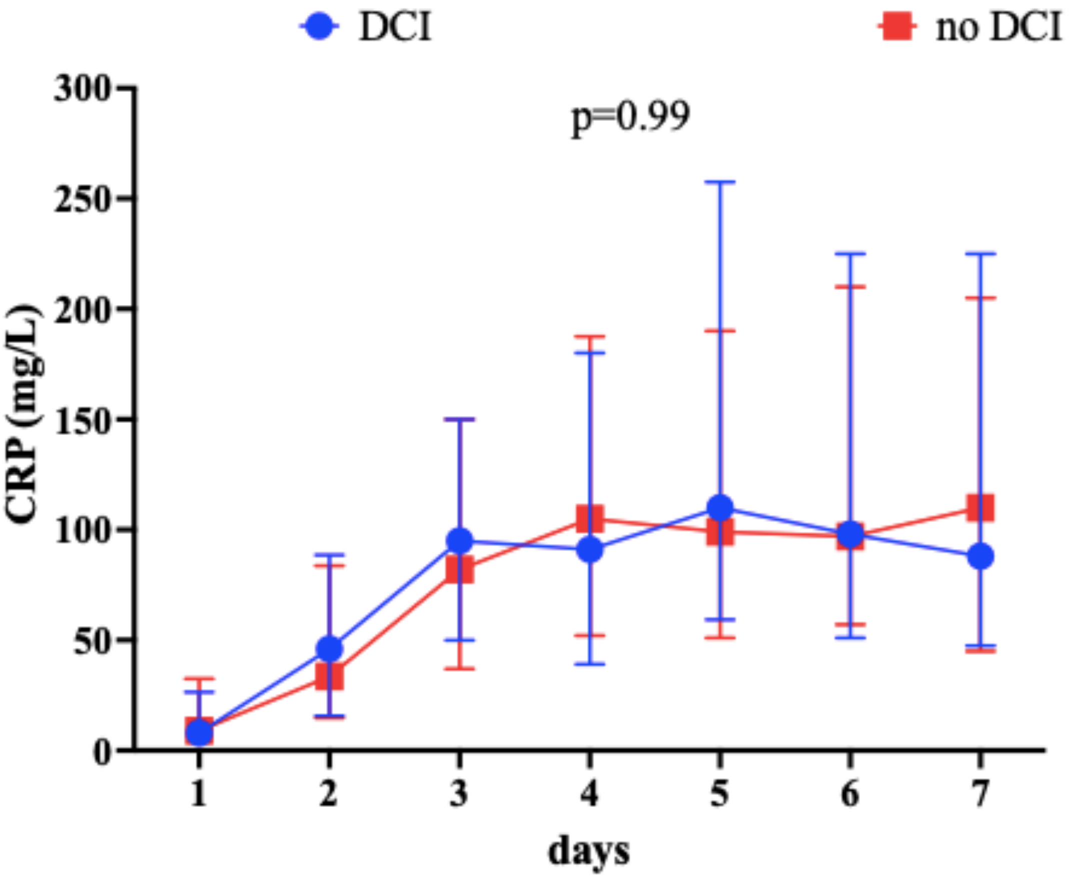

B)

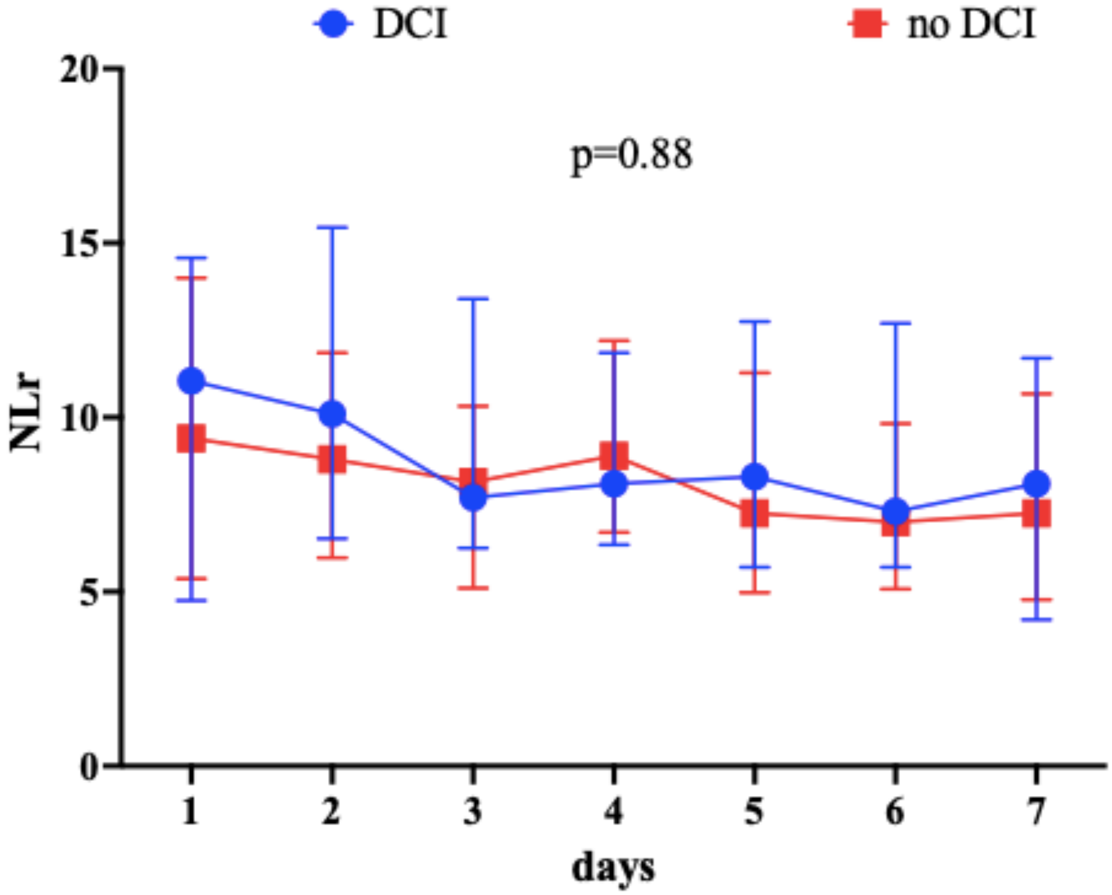
DCI
no DCI
p=0.88
NLr
20
15
10
5
0
1
2
3
4
5
6
7
days
